# Extracting Evolutionary Insights Using Bioinformatics

**DOI:** 10.1155/2013/376235

**Published:** 2013-11-13

**Authors:** Dmitry Sherbakov, Yuri Panchin, Ancha Baranova

**Affiliations:** ^1^Laboratory of Molecular Systematics, Limnological institute, Ulan-Batorskaya Str. 3, Irkutsk 664033, Russia; ^2^Department of Biology, Irkutsk University, Sukhe-Bator 5, Irkutsk 664003, Russia; ^3^Department of Mathematical Methods in Biology, Belozersky Institute, Moscow State University, Vorbyevy Gory 1-40, Moscow 119991, Russia; ^4^Institute for Information Transmission Problems, Russian Academy of Sciences, Bolshoi Karetny Pereulok 19-1, Moscow 127994, Russia; ^5^Research Centre for Medical Genetics, Russian Academy of Medical Sciences, Moscow 115478, Russia; ^6^School of System Biology, George Mason University, Fairfax, VA 22030, USA

According to much quoted Russian/American geneticist Theodosius Dobzhansky, “*Nothing in biology makes sense except in the light of evolution*” [1]. Coined in 1973, this famous phrase remains up to date. Fascinating variety of the phenotypes seen as either visible or subtle differences in species, populations, and individual organisms inhabiting our planet attracts an unwavering inquiry. The results of these inquiries are shedding some light both at intricate molecular programs continuously executed within living bodies and at checks and balances within homeostatic ecosystems. In fact, every transformative technology so far invented to improve human medicine or other areas of applied science was immediately embraced by traditional biologists and contributed to the extraction of evolutionary insights. Some years ago, that happened with PCR. Recently, we entered systems biology driven turn of an inquiry spiral. This turn is enabled by an advent of NextGen sequencing. Importantly, a few final steps on this spiral became computationally heavy. Hence, the aid of bioinformatics was summoned, and computationally derived conclusions came in droves. 

While the mining of completed genomes remains an obvious source is information on evolutionary processes, we also see the emergence of a novel kind of the full-genome studies, ones that aim at the tinkering with the evolutionary theory itself. By their scale and potential, these studies may be compared to the two previous technological developments that changed the history of evolutionary studies. The first of these methodological revolutions is a comparative analysis of traits that was started by Lamarck [2] and Darwin [3] and still is a source of novel evolutionary inferences. The second one was the introduction of the molecular analysis (see [4]) that approximately coincided with the establishment of the synthetic theory of evolution. Since that, the methods of the analysis of molecular traits kept progressing. By now, it is clear that molecular-based inquiry opened whole new field of questions which may be asked and answered, for example, one that relates to the evolution of the intracellular parasites, microbial communities, or populations of the cells within human body burdened with a tumor. The introduction of NextGen sequencing dramatically increased the amount of information that may be extracted from the sample of biological material and added the whole new level of integration of organismal data, thus enabling the systems biology in its true sense. 

Nowadays, one may be certain that widespread adoption of NextGen sequencing will uplift the evolutionary theory to new heights, however, where will it bring us even in next few years is substantially harder to predict. We should not forget that evolutionary genomics is still an emerging field in biology. For now, it does not have a road map, a master plan, or even a template—one who starts the analysis of data collected from the study of living system never knows where this analysis might end. This volume is yet another attempt to provide a bird's view at bioinformatics-driven forays into the field of evolutionary biology. 

However, it is an opinion of the Editors that presented eclectic collection of papers is not without its merits. In case of existing data, even a mere push to extract an evolutionary insight enables the scientist to see the pattern that would be very difficult to discern otherwise. So much better it is for the studies specifically designed to solve one or another biological enigma. In this issue, we are glad to present an eclectic collection of papers that cover a range of topics from analysis of intron evolution across kingdoms to the study of the divergence in salmonid species and to comparative genomics of cancer-specific genes. These manuscripts are a sampler of future insights yet to come from the troves of genome sequences thoroughly dissected with bioinformatics tools. Enjoy!

*Dmitry Sherbakov* *Dmitry Sherbakov* *Yuri Panchin* *Yuri Panchin* *Ancha Baranova* *Ancha Baranova*

## References

[1] Dobzhansky T (1973). Nothing in biology makes sense except in the light of evolution. *American Biology Teacher*.

[2] Lamarck JB (1914). *Zoological Philosophy*.

[3] Darwin C (1859). *On the Origin of Species by Means of Natural Selection, Or the Preservation of Favoured Races in the Struggle for Life*.

[4] Avise JC (2004). *Molecular Markers, Natural History and Evolution*.

